# Extra-foraminal Intraneural L5-S1 Disc Herniation Mimicking a Retroperitoneal Peripheral Nerve Sheath Tumour: Case Report and Review of the Literature

**DOI:** 10.7759/cureus.4956

**Published:** 2019-06-20

**Authors:** Yigit Ozpeynirci, Michael Braun, Inga Lubotzki, Bernd Schmitz, Gregor Antoniadis

**Affiliations:** 1 Neuroradiology, Ulm University, Ulm, DEU; 2 Neurosurgery, Ulm University, Ulm, DEU; 3 Radiology, Ulm University, Ulm, DEU

**Keywords:** disc herniation, extra-foraminal, pre-sacral mass, mri

## Abstract

Disc herniations can present with unusual findings at unusual locations, mimic different pathologies and create confusion in the daily practice. Extra-foraminal intraneural location of L5-S1 disc herniation is extremely rare and may not be noticed on initial imaging extending the time to reach the diagnosis. There is no specific imaging finding suggesting the intraneural location of the lesion. Here, we report a case of an extra-foraminal intraneural L5-S1 disc herniation mimicking a retroperitoneal peripheral nerve sheath tumour and review similar cases in the literature.

## Introduction

Disc herniation, seen at lumbar levels commonly in the ventral epidural space, can present with unusual findings at unusual locations, mimic different pathologies and create confusion in the daily practice. Here, we report a case of an extra-foraminal L5-S1 disc herniation mimicking a retroperitoneal peripheral nerve sheath tumour and discuss similar cases in the literature.

## Case presentation

**Figure 1:**
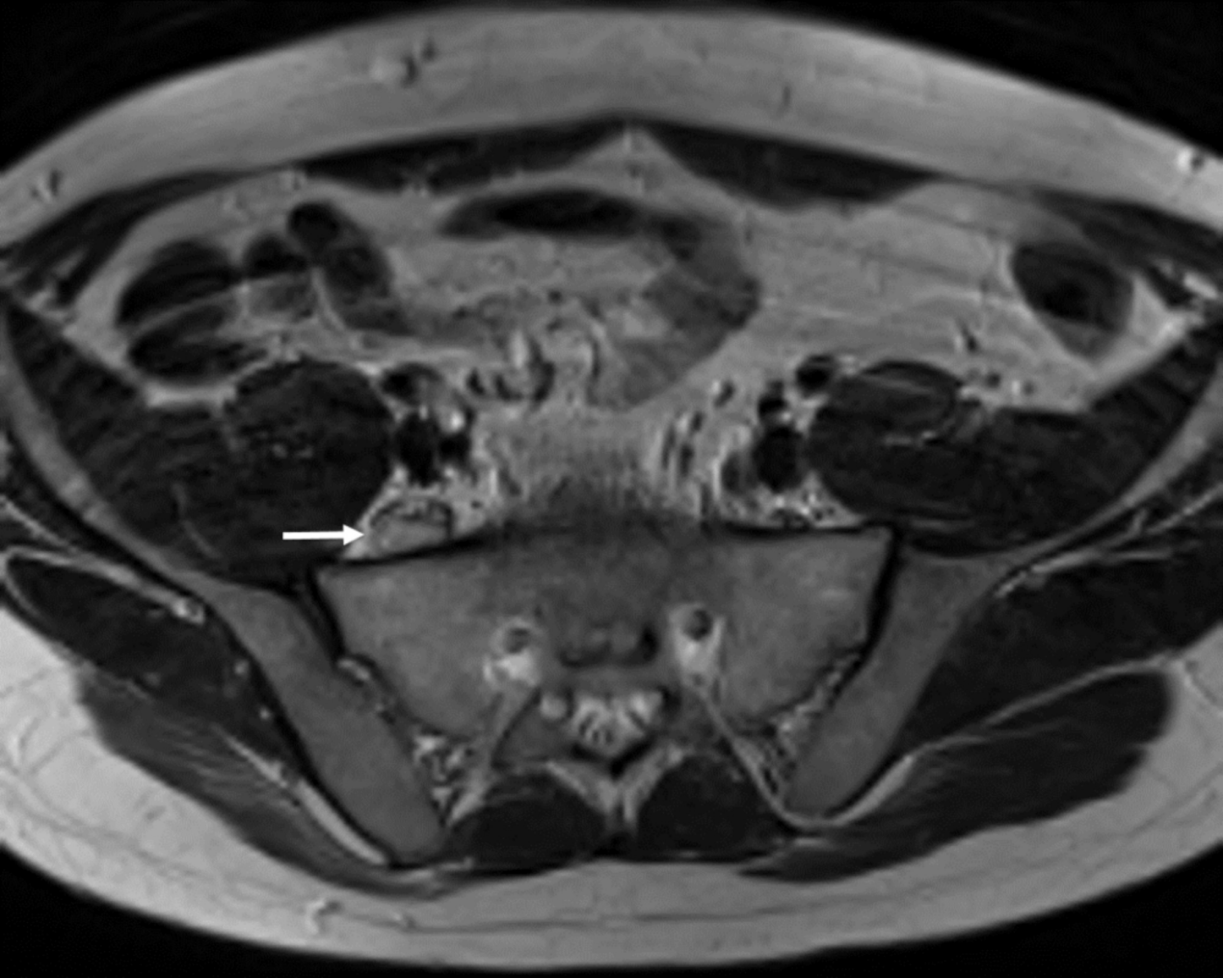
Right pre-sacral mass Axial T2 weighted magnetic resonance imaging (MRI). Retroperitoneal right pre-sacral mass (white arrow) measuring 18 x 8 x 30 mm and displacing the nerve fibres anteromedially suggesting a nerve sheath tumour

**Figure 2:**
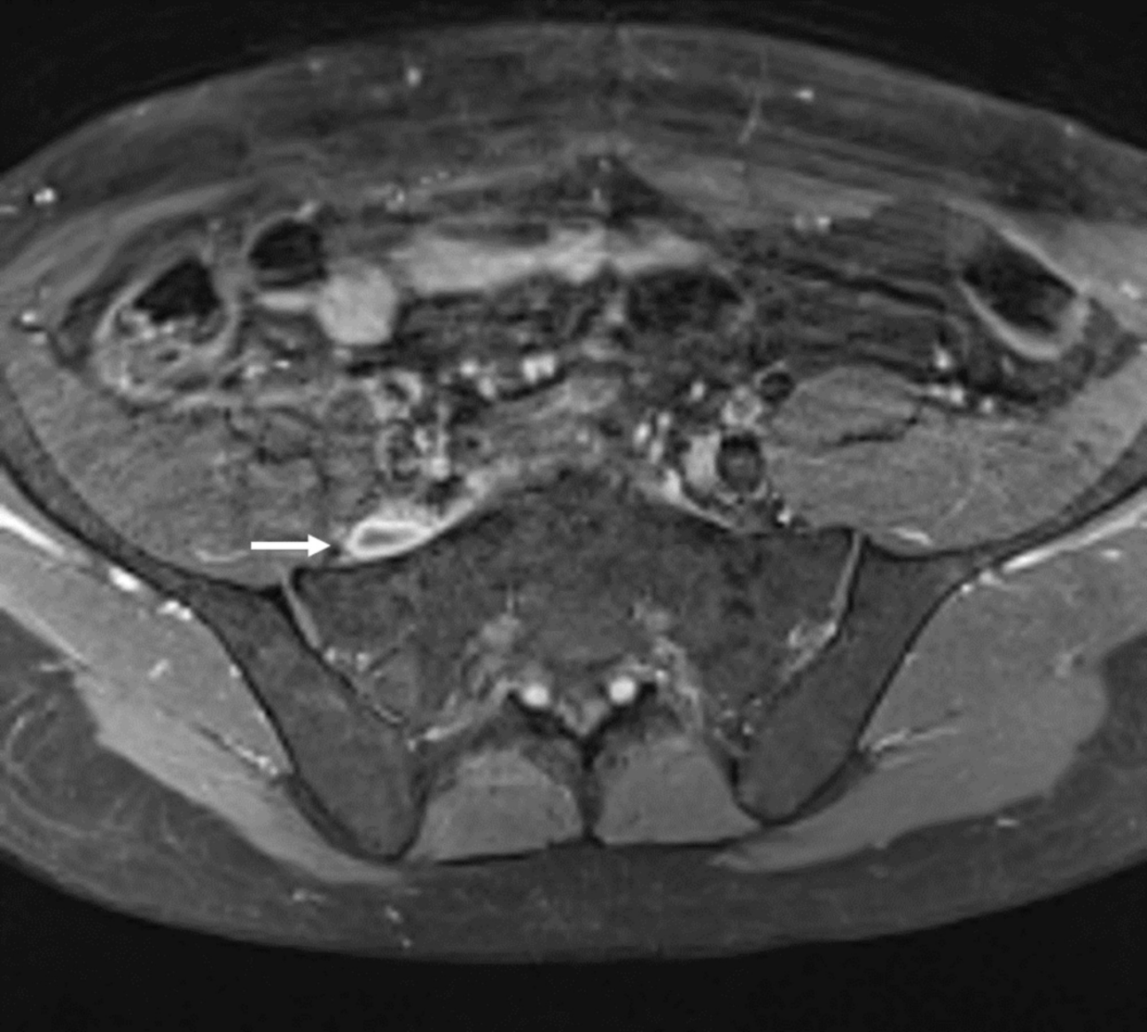
Right pre-sacral mass Axial fat suppressed contrast-enhanced T1 weighted magnetic resonance imaging (MRI) showing peripheral contrast enhancement of the right pre-sacral mass (white arrow)

**Figure 3:**
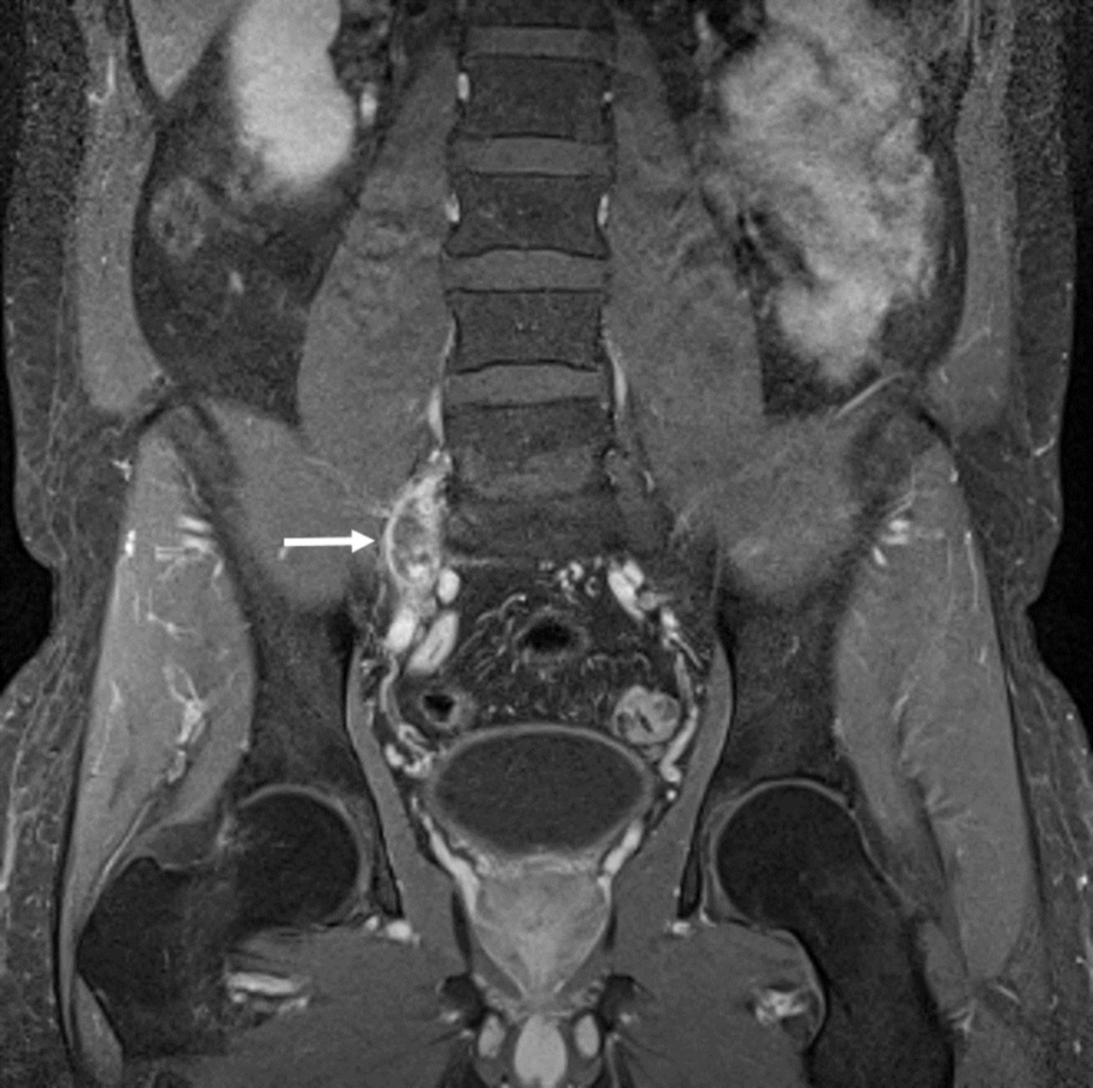
Right pre-sacral mass Coronal fat suppressed contrast-enhanced T1 weighted magnetic resonance imaging (MRI) demonstrating peripheral contrast enhancement of the right pre-sacral mass (white arrow)

A 42-year-old otherwise healthy man presented to another hospital with sudden right-sided radicular leg pain corresponding to the L5 dermatome. Lumbar computed tomography (CT) and magnetic resonance imaging (MRI) were reported normal. After persistence of the pain for nearly a month and acute development of foot drop, he was admitted again. Pelvic MRI showed a retroperitoneal pre-sacral mass, misdiagnosed on initial images, suggesting a nerve sheath tumour compressing the right L5 nerve (Figure 1-3).

**Figure 4:**
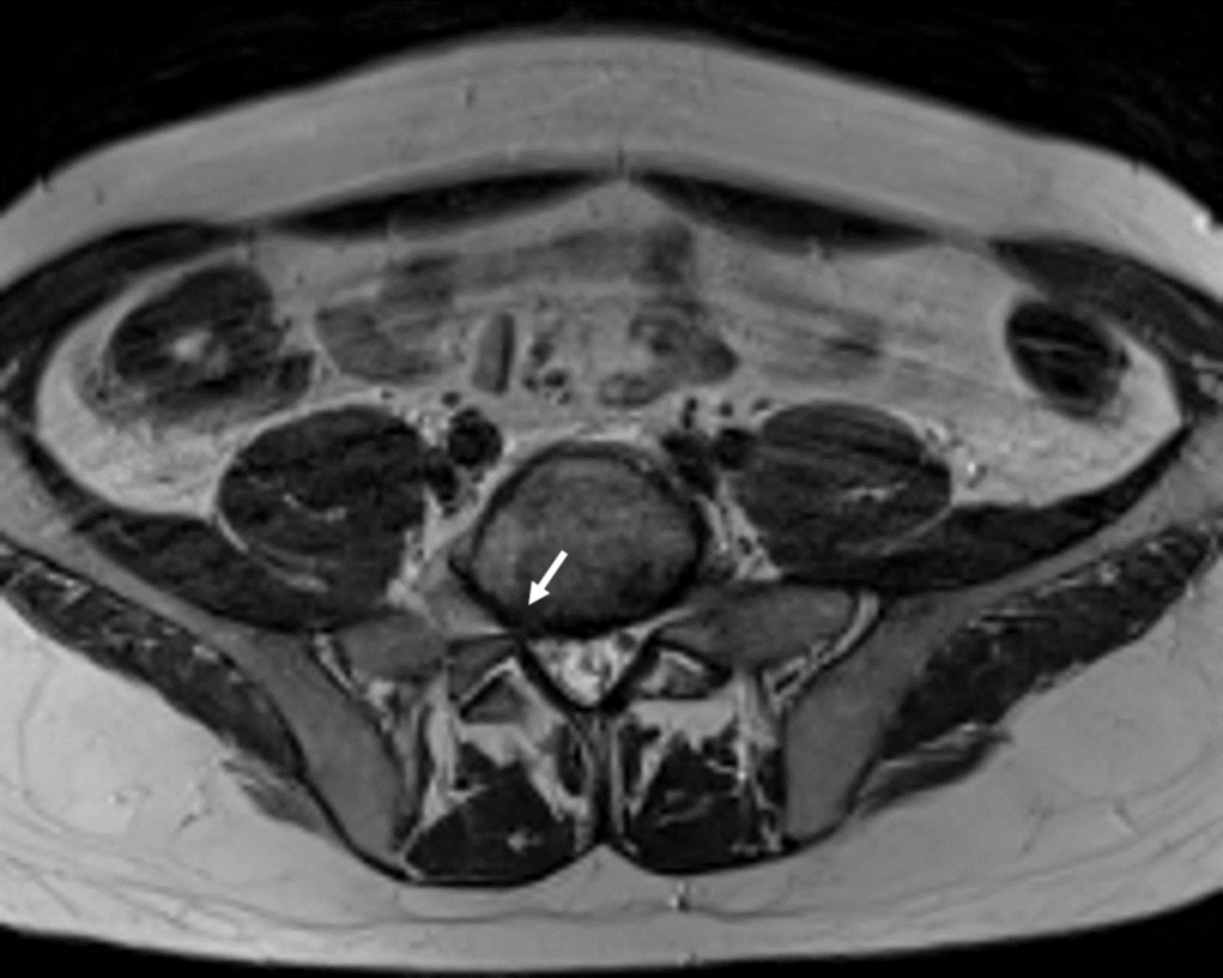
L5/S1 disc level Axial T2 weighted magnetic resonance imaging (MRI) showing L5-S1 disc bulging without significant mass effect (white arrow)

He then was referred to our clinic for further treatment and eventual surgery. On admission, the visual analog scale pain score was eight. Neurological examination demonstrated a positive Lasegue sign, 4/5 paresis of the dorsiflexors of the foot and 3/5 paresis of the dorsiflexors of the great toe, dysesthesia and vegetative changes (progressive oedema and colour changes) on L5 sensory dermatome and normal lower extremity reflexes. MRI showed a right pre-sacral mass adjacent to the right L5 nerve measuring 18 x 8 x 30 mm and displacing the nerve fibres anteromedially. The mass was isointense to the skeletal muscle on T1-weighted and hyperintense on T2-weighted images and had peripheral contrast enhancement (Figure 1-3). The L5-S1 intervertebral segment showed mild degeneration and a slightly right-sided disc bulging without any significant mass effect (Figure 4).

**Figure 5:**
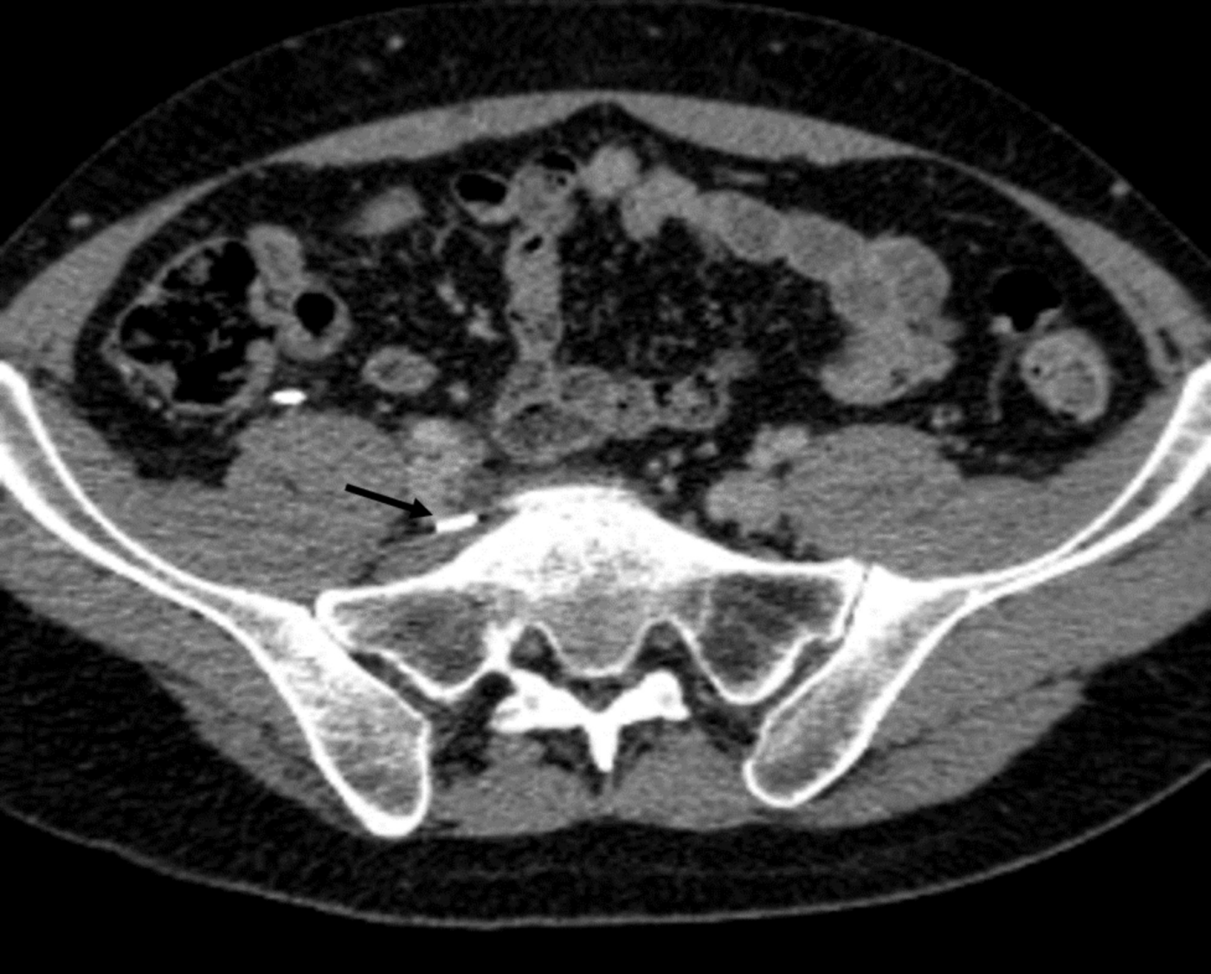
Post-operative CT Axial computed tomography (CT) demonstrating metallic clip anterior to the mass (black arrow)

A caudally migrated L5-S1 disc herniation was suspected but a nerve sheath tumour could not be ruled out. A repeat MRI in our institution one month after the first one confirmed the mass lesion and showed no interval change of imaging findings. Percutaneous periradicular infiltration of the L5 nerve with local anesthetics and steroids at the intra-foraminal level caused a significant but temporary pain relief. An attempt to remove the mass surgically through a laparoscopic anterior abdominal approach failed because the tumour could not be identified and the surgical bed was marked with a clip which was located just anterior and medial to the mass (Figure 5).

**Figure 6:**
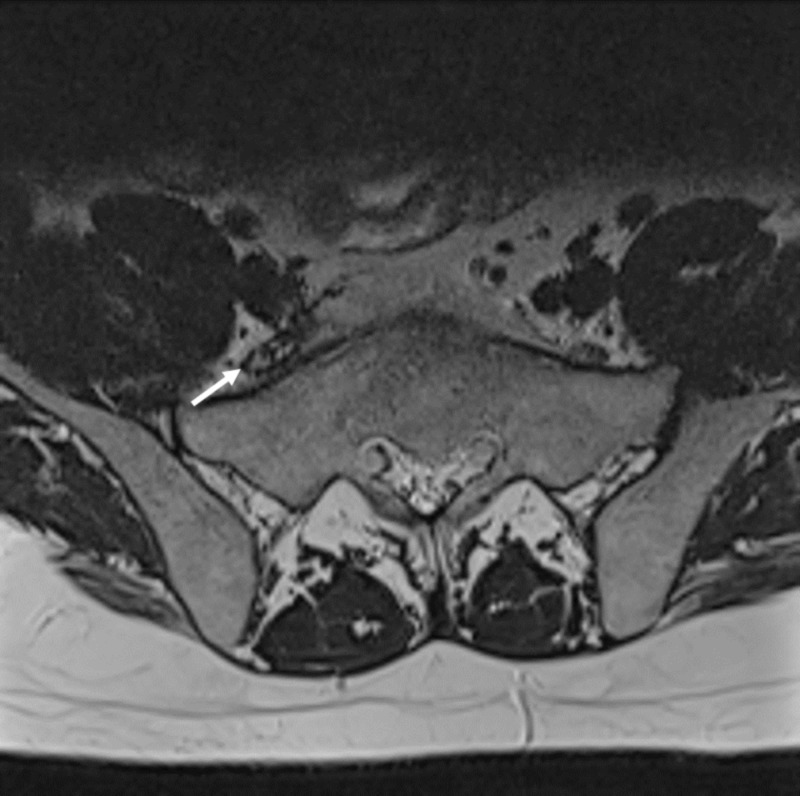
Post-operative magnetic resonance imaging Axial T2-weighted magnetic resonance imaging (MRI). Post-operative MRI showing adequate decompression and a hemosiderin rim surrounding the nerve (white arrow)

At the second attempt, the mass could be fully removed through a microsurgical approach following a lower abdominal laparotomy and an epineurotomy. Thus, the intraneural location of the lesion could be confirmed. Intra-operative biopsy ruled out a tumour and confirmed the diagnosis of disc herniation. Post-operatively, the patient reported significant improvement of his complaints but hypaesthesia on the dorsum of the foot persisted. The patient had an uneventful post-operative recovery except a transient paralytic ileus treated conservatively, and he could be discharged home six days after the second operation. A post-operative MRI showed adequate decompression and a hemosiderin rim surrounding the nerve (Figure 6).

## Discussion

We report on an extra-foraminal intraneural L5-S1 disc herniation in the pre-sacral region mimicking a retroperitoneal peripheral nerve sheath tumour that was missed on initial MRI. Because of persistent pain and development of foot drop, re-evaluation of the images and a pelvic MRI confirmed the mass lesion. Intraneural migration of extra-foraminal herniated disc material is extremely rare and to our knowledge, it has been reported only once before [1].

Extra-foraminal disc herniation is an underdiagnosed entity [1-2] with L4-5 being the most commonly involved level (38%) [3]. Osborn et al. reported that one-third of extra-foraminal herniations were initially not diagnosed accurately. In their series, nearly 20% of extra-foraminal disc herniations were exclusively lateral to the foramen without an intra-foraminal component [3]. The diagnosis becomes more complicated, especially when sequestered herniation occurs at some distance from the foramen. The standard imaging technique is MRI. Signal characteristics of herniated discs are variable. Contrast enhancement is typically present and expected to be rim-type as seen in our case, although a more homogenous enhancement pattern can be seen, especially in cases of long-standing disc herniation [1,4-5]. Imaging and clinical differential diagnosis of extra-foraminal disc herniations may include abscesses, metastases or primary tumours, such as benign or malignant nerve sheath tumours. To rule out an abscess or a metastasis, patient’s history and laboratory diagnostics would be helpful. On MRI, peripheral nerve sheath tumors are mostly well circumscribed fusiform-shaped masses with tapered ends displacing the adjacent structures without invasion. They usually show intense enhancement and may demonstrate remodeling of the adjacent bony structures best appreciated on CT. However, heterogenous appearance due to cystic and fatty degeneration, hemorrhage or calcification can occur. Although differentiation based on imaging is not reliable, bigger lesion size, irregular borders, infiltration of nearby structures and rapid growth in interval imaging are more typical for malignancy [6-7]. Sometimes multiple variable imaging techniques (CT and MRI of lumbar spine or pelvis, myelogram, positron-emission tomography (PET) scan, discography, bone survey or CT of the chest and abdomen) are employed to reach a diagnosis and exclude other possible aetiologies. Our patient had neither a history of cancer nor laboratory results indicating an infectious disease. Sudden onset of symptoms and the rim-type enhancement of the lesion made us think of a disc herniation.

Caudal migration of extra-foraminal disc herniations on the L5-S1 level may present as a retroperitoneal mass. A literature survey revealed only four other cases [1,4-5,8]. They all presented with a pre-sacral mass far from the intervertebral foramen showing anterior displacement of the L5 root and symptoms typical for an L5 radiculopathy. Table 1 summarizes the characteristics of other cases. In two of those cases, the mass was initially not seen just as in our case [5,8]. In nearly all of the cases, either initial imaging was repeated or multiple imaging studies were done to exclude other possible pathologies before surgery. The lesions were removed either through anterior or posterior surgical approaches. In one case, after the removal of the herniated disc material the symptoms worsened and the remaining intra-foraminal disc herniation at the same level was removed via a posterior approach [1].

**Table 1:** Characteristics of cases with extra-foraminal disc herniations on L5-S1 level presenting as a pre-sacral mass CT: computed tomography, MRI: magnetic resonance imaging, PET: positron-emission tomography

Authors	Patient age and sex	Pre-operative differential diagnoses	Detected on initial imaging	Pre-operative imaging	Surgical approach	Follow-up
Tschugg et al. [5]	33-year-old man	Herniated disc, schwannoma	No	Lumbar CT, MRI and repeat lumbar MRI	Posterior approach (lateral extraforaminal transmuscular approach)	Full recovery (one year)
Sharma et al. [1]	55-year-old man	Malignant peripheral nerve sheath tumor	Unknown	Lumbar CT and MRI, whole-body PET, metastatic bone survey, CT of chest and abdomen, repeat lumbar MRI	First surgery: transabdominal transperitoneal route Second surgery: posterior route	Partial recovery (18 months)
Levene et al. [4]	76-year-old woman	Nerve sheath tumor	Unknown	Lumbar MRI (not clearly described)	Anterior retroperitoneal approach	Partial recovery (12 weeks)
Perves et al. [8]	47 year-old man	Herniated disc	No	Lumbar CT, myelogram, discography-CT	Anterior transperitoneal approach	Full recovery (one month)
Özpeynirci et al.	42-year-old man	Herniated disc suspected, schwannoma	No	Lumbar MRI, pelvic MRI, lumbar repeat MRI and lumbar CT	Anterior transperitoneal approach (both surgeries)	Partial recovery (1 year)

Intraneural location of the sequester made our case more interesting. Intradural location of a disc herniation is a rare, but well-known entity [9]. However, intraneural migration of the herniated disc material is extremely rare and has been reported only once before [1]. It has been hypothesized that the herniated disc can damage the epineurium and infiltrate the nerve [1,10]. There is no reliable imaging finding to predict the intraneural location of disc material before surgery. Sharma et al. reported anterior and slightly medial displacement of the L5 nerve just as in our case. Aside from other multiple imaging studies, they did a pre-operative PET which showed mildly increased fluorodeoxyglucose uptake suggesting a malignant peripheral nerve sheath tumour.

## Conclusions

Extra-foraminal intraneural L5-S1 disc herniation presenting as a pre-sacral mass is extremely rare and may not be noticed on initial imaging extending the time to reach the diagnosis. Careful examination of the extra-foraminal area is thus recommended, especially if no intraspinal finding corresponds to the clinical presentation.
